# Genistein From *Fructus sophorae* Protects Mice From Radiation-Induced Intestinal Injury

**DOI:** 10.3389/fphar.2021.655652

**Published:** 2021-05-21

**Authors:** Jieyu Zhang, Zhijun Pang, Yuting Zhang, Jiaxin Liu, Zhaowei Wang, Chuanyang Xu, Lei He, Weina Li, Kuo Zhang, Wangqian Zhang, Shuning Wang, Cun Zhang, Qiang Hao, Yingqi Zhang, Meng Li, Zhengmin Li

**Affiliations:** ^1^ The State Key Laboratory of Cancer Biology, Department of Biopharmaceutics, School of Pharmacy, Fourth Military Medical University, Xi’an, China; ^2^ Department of Dermatology, Xijing Hospital, Fourth Military Medical University, Xi’an, China; ^3^ Department of Laboratory Medicine, The 971th Naval Hospital, Shandong, China; ^4^ Second Battalion of Basic Medical College, Fourth Military Medical University, Xi’an, China; ^5^ Department of Anesthesiology, Tangdu Hospital, Fourth Military Medical University, Xi’an, China

**Keywords:** *Fructus sophorae*, X-ray irradiation, iirradiation-induced intestinal injury, active pharmaceutical ingredient, genistein

## Abstract

The development of an effective pharmacological countermeasure is needed to reduce the morbidity and mortality in high-dose ionizing radiation-induced acute damage. Genistein has shown bioactivity in alleviating radiation damage and is currently synthesized by chemosynthetic methods. Due to concerns about chemical residues and high costs, the clinical application of genistein is still a major challenge. In this study, we aimed to establish an efficient method for the extraction of genistein from *Fructus sophorae*. The effects of extracted genistein (FSGen) on preventing intestinal injury from radiation were further investigated in this study. C57/BL mice were exposed to 7.5 Gy whole body irradiation with and without FSGen treatments. Histological analysis demonstrated significant structural and functional restitution of the intestine and bone marrow in FSGen-pretreated cohorts after irradiation. Through mRNA expression, protein expression, and small interfering RNA analyses, we demonstrated that FSGen protects IEC-6 cells against radiation damage by upregulating the *Rassf1a* and *Ercc1* genes to effectively attenuate DNA irradiation damage. Together, our data established an effective method to extract genistein from the *Fructus sophorae* plant with high purity, and validated the beneficial roles of the FSGen in protecting the radiation damage. These results promise the future applications of *Fructus sophorae* extracted genistein in the protection of radiation related damages.

## Introduction

Radiotherapy is considered the most effective cancer treatment after surgery, but it can cause damage to normal tissue and organs, especially digestive tract epithelium and hematopoietic tissue (Schaue and McBride, 2015). Ionizing radiation-induced (IR-induced) damage to the gastrointestinal (GI) tract is a frequent side effect of radiation therapy, and a considerable proportion of patients experience acute or chronic diarrhea, abdominal pain, and nausea, among other symptoms (Tabibian et al., 2017). Approaches to maximize the radiation dose administered to abnormal cancer cells while minimizing injury to normal cells and alleviating the side effects of radiation are the key issues in radiotherapy.

Genistein is a soy-derived isoflavonoid compound with a multitude of health benefits. Various studies have suggested that genistein exerts radioprotective activities by protecting against DNA damage and by scavenging free radicals, such as hydroxyl radicals (Landauer et al., 2003; Wei et al., 2003). The Ersoz S. group demonstrated that genistein has radioprotective effect on mouse liver injury induced by whole-body irradiation (WBI) (Hanedan Uslu et al., 2019). The Son group suggested that genistein had a protective effect on the intestinal damage induced by irradiation and that it delayed tumor growth (Son et al., 2013). Moreover, genistein showed an effective radioprotective activity against hematopoietic-acute radiation syndrome in a murine model (Zhou and Mi, 2005; Davis et al., 2008; Ha et al., 2013; Landauer et al., 2019). It was further confirmed that genistein possessed radio-sensitizing effect toward tumor cells but radioprotective effect against normal cells (Kim et al., 2011; Kim et al., 2014).

Although these results suggest that genistein is a useful candidate for preventing radiotherapy-induced damage in cancer patients, its drug manufacturing and molecular mechanism still require further exploration. Currently, BIO 300, a patented nanoparticle formulation of genistein, has United States FDA investigational new drug status (Kamran et al., 2016). BIO 300 significantly increases mouse survival and improves hematopoietic response and lung injury against a lethal dose of radiation when injected subcutaneously and orally (Jones et al., 2017). While BIO 300 was safe and well-tolerated when administered orally for 14 days in healthy volunteers in phase-I trials (Zenk, 2007), concerns regarding chemical residues and high costs are still barriers to its clinical application.

*Styphnolobium japonicum* (L.) Schott [syn.: *Sophora japonica* L.; Fabaceae]., known as Huai in Chinese, which is commonly found in China, Japan, Korea, Vietnam, and other countries, is a shrub belonging to the subfamily Faboideae of the Fabaceae. *Fructus sophorae* is commonly used in TCM., is used as medicine in Asia to cool the blood and stop bleeding (Lao et al., 2005). Previous phytochemical investigations revealed that genistein, kaempferol, quercetin, rutin, isorhamnetin, and sophoricoside are the major active constituents of *S. japonica* (He et al., 2016). In this study, we established genistein extraction method by using batch solvent extraction, and producing FSGen with high purity and at a low cost for use as an active pharmaceutical ingredient (API). Furthermore, to elucidate the medicinal activity of extracted genistein, we evaluated the protective effect of FSGen on IR-induced intestinal damage in a cell model and in mouse models exposed to X-ray irradiation. Our results indicated the genistein from *Fructus sophorae* have a role in protecting the radiation damage and this effect may be mediated mainly by increasing the DNA damage repair genes expression, especially *Rassf1a* and *Ercc1*. Since genistein is one of the major active constituents of Fructus sophorae, establish the extracting method is important to promote the industrialization of *Fructus sophorae*.

## Materials and Methods

### Plant Material

The plant name, synonym of *Styphnolobium japonicum* (L.) Schott., were confirmed with the Medicinal Plant Names Services (http://mpns.kew.org). *Fructus sophorae* (Huai Jiao) is the dried ripe fruit of *Styphnolobium japonicum* (L.). *Fructus sophorae* was purchased from the Shaanxi pharmaceutical wholesale market, and identification and collection were performed according to the Pharmacopoeia of China (2000). An authentic sample of the collection was preserved in the Department of Biopharmaceutics at Fourth Military Medical University by register number 槐20170401.

### Genistein Extraction

A 10 g dried sample of *Fructus sophorae* was powdered, and genistein was extracted from the sample by reflux extraction (pH 8.5, hot alkaline solution) with a liquid-solid ratio (g/ml) of 1:4, 1:3, and 1:2 at 96–99°C for 2.5, 2, and 1.5 h, respectively. Subsequently, the solution was collected, and a quantity of concentrated hydrochloric acid was added for 2.5 h of reflux extraction at 95°C for sophoricoside acidolysis. The extract was diluted 10-fold with water and centrifuged 6,000 × g for 25 min to collect the pellet. EtOH was added to the pellets, followed by reflow discoloration with activated carbon and pulp filtration. The supernatant was concentrated by rotary evaporation and powdered by a spray dryer.

The concentrations of extract were calculated using the corresponding standard genistein curve. The experiment was performed in triplicate.

### FSGen Analytical Method

FSGen was identified by high-performance liquid chromatography (HPLC) and mass spectrometry (MS). HPLC was performed using a water/methanol (40:60) system and equipped with Diamonis/TM C18 pumps at a flow rate of 1.2 ml/min. The detection was performed at a wavelength of 260 nm (Gold System HPLC, Beckman Coulter, United States). MS was performed using an HP 5989B mass spectrometer (Agilent, United States). Furthermore, the structure of FSGen was confirmed by spectroscopic evidence. UV spectra were recorded on a UV-2100 spectrophotometer (Shimadzu, Japan) at a wavelength of 190–700 nm. The infrared spectroscopy (IR) spectra were measured on a Nicolet 60SXR spectrometer (Thermo Fisher scientific, United States). Nuclear magnetic resonance spectroscopy (NMR) spectra were obtained on INOVA-400 (Varian, Japan) (500 MHz for ^1^H; 125 MHz for ^13^C) in DMSO-*d6* as solvents.

### Cell Culture and Treatment

The rat small intestine IEC-6 cell line was purchased from Shanghai Zhong Qiao Xin Zhou Biotechnology Co., Ltd. (Shanghai, China). Cells were cultured in DMEM (Gibco-Invitrogen, United States) supplemented with 10% FBS and penicillin/streptomycin at 37°C under 5% CO_2_. For all experiments, cells in the exponential phase of growth were used. Different concentrations of FSGen (0, 2, 4, 8, and 16 μmol/L) were applied to the cells for 48 h, and then the appropriate concentration of FSGen was determined by CCK-8 assay. Cells were treated with different concentrations of FSGen (0, 2, 4, 8, and 16 μmol/L) followed by irradiated with different doses of X rays (6 and 12 Gy) at a dose rate of 444.4 cGy/min (RS2000 X-ray biological irradiator (Rad Source Technologies, Inc., Boca Raton, United States) and were harvested 24, 48, and 72 h after irradiation. The irradiation dose was determined by a cell viability assay, and 6 Gy of irradiation was eventually selected in the following experiment.

To study the radioprotective effect of FSGen, the cells were divided into a nontreated control (Normal) group, vehicle (DMSO) + X-ray irradiation group (IR), and FSGen + irradiation (FSGen + IR) group. The 4 μmol/L of FSGen was applied in the FSGen treatment group 24 h prior to irradiation.

### Cell Viability Assay

Cell viability was evaluated using the CCK-8 assay. Briefly, after the administration of treatments, the cells were treated with 10 μL of CCK-8 (Biosharp, China) and incubated for 1 h at 37°C. The absorbance was read at 450 nm on an ELX800 microplate reader (Biotek, United States). The experiment was performed in triplicate at a minimum.

### Cell Apoptosis Assay, Senescence Assay, and Reactive Oxygen Species Assay

Vehicle (DMSO) or 4 μM FSGen was added to IEC-6 cells for 24 h before 6 Gy of X-ray irradiation. The cells were cultured for 48 h after irradiation and then prepared for further analysis. DMSO-treated cells and normal cells were used as controls. All experiments were performed in triplicate at a minimum.

For the apoptosis assay, the cells were collected, stained with V-FITC and propidium iodide (PI), and detected by flow cytometry (Bio-Rad, United States). For the senescence-associated β-galactosidase (SA-β-gal) staining assay, the cells were fixed, washed with PBS, and then stained with SA-β-gal staining solution (Solarbio, China) at 37°C overnight in a dry incubator. The development of the blue color of the cells was checked under a microscope (Nikon, Japan). For the ROS assay, the cells were washed with PBS and then cultured with DMEM containing DCFH-DA (Solarbio, China) for 30 min at 37°C. The cells were carefully washed twice with PBS, and the cells were observed under a confocal microscope (Ex/Em = 500/525 nm) (LSM 900, ZEISS, Germany).

### Single-Cell Gel Electrophoresis Assay (Comet Assay)

FSGen (4 μmol/L) was added to cultured IEC-6 cells for 24 h before 6 Gy of X-ray irradiation was administered. At 48 h after irradiation, the cells were harvested and suspended in PBS for the SCGE comet assay (Merck, United States), which was performed to determine the degree of DNA damage. Briefly, frosted microscope slides were covered with 80 μL of 1% normal melting agarose (NMA) at 45°C and immediately stored at 4°C for 10 min to allow the agarose to solidify. Then, a second cover layer consisting of 75 μL of 0.8% low melting agarose (LMA) and approximately 1 × 10^3^ cells at 37°C was added, and the slides were immediately covered and stored at 4°C for 10 min. Then, 85 μL of 0.5% LMA was added at 37°C to the second LMA layer and stored at 4°C to allow the agarose to solidify. After the LMA solidified, the slides were removed and placed in a lysing solution for 1 h at 4°C to remove the protein and RNA from the nuclear scaffold. The slides were washed and placed in a horizontal electrophoresis tank filled with an alkaline buffer for DNA electrophoresis. After 30 min of electrophoresis at 25 V, the slides were washed with Tris-HCl (pH 7.5) for 15 min and then stained with 50 μL of ethidium bromide (EB) for 20 min in the dark. The slides were visualized under a fluorescence microscope (Nikon, Japan). The DNA strand breaks were quantified with stored images using CASPLab software, and the tail moment is defined as the product of the tail length and the fraction of total DNA in the tail. Olive Tail Moment = Tail DNA% x Tail Moment Length. The experiment was performed in triplicate at a minimum.

### Mouse Experiments

The animal studies were approved by the institutional review board (20180106), and carried out in accordance with the National Institutes of Health guide for the care and use of laboratory animals. Female C57BL/6 mice at 8 weeks of age were obtained from the Animal Experiment Center of the Fourth Military Medical University (Xi’an, China) and housed in a specific pathogen-free animal facility.

We first conducted the pilot study to determine the dose of X rays. 15 mice were randomly divided into three groups and were exposed to WBI at 3.5, 7.5, and 11.5 Gy of X-rays (Rad Source Technologies, Inc. Boca Raton, United States). The irradiation dose was determined by the survival probability at three-time points (days 0, 10, 20) of different groups. To evaluate the effect of FSGen on radiotherapy protection, 50 mice were randomly divided into five groups as follows: A, FSGen + irradiation; B, vehicle + irradiation; C, FSGen + sham irradiation; D, genistein + irradiation, and E, vehicle + sham irradiation. The vehicle was referred to polyethylene glycol 400 (PEG400 (Sigma-Aldrich, United States). PEG400, is highly valued in pharmaceutical applications due to its low toxicity and its exceptional ability to solubilize polar active pharmaceutical ingredients. FSGen was dissolved in PEG400 on the day of the experiment by sonication for 20 s (Heat Systems-Ultrasonics Inc.). FSGen, genistein (Sigma-Aldrich, United States) and vehicle were given by subcutaneous (sub-Q) injection on the day before irradiation (200 mg/kg, 0.1 ml/mouse). The dose of FSGen was considered optimum for radioprotection, as reported previously (Landauer et al., 2003). WBI was administered at a dose rate of 222.2 cGy/min by using an RS2000 X-ray biological irradiator (Rad Source Technologies). After irradiation, three mice were euthanized randomly in each group at 8 days for following analysis, while others recorded the death time and number of deaths for 30 days for survival analysis.

### Apoptosis Assay

After irradiation, the mouse sterna were collected and fixed in formaldehyde for at least 24 h. Samples were decalcified and sectioned longitudinally for subsequent staining. Additionally, the entire small intestine was harvested, and a 1 cm duodenal sample was fixed for histology in 4% formalin for 24 h and embedded in paraffin. Hematoxylin-eosin (H&E) and TUNEL (*In Situ* Cell Death Detection Kit, Fluorescein) analysis were used to score apoptosis in prepared paraffin sections in seven mice from each group. H&E staining was performed on 4-μm-thick paraffin sections. Images of staining sections were obtained by Aperio Digital Pathology Scanner (Aperio, United States). Three images of each section were randomly selected, and the height of intestinal villus and blank area of sterna was measured in NDP. View 2 software (Hamamtsu, Japan).

The TUNEL assay was carried out according to the kit instructions (Roche, United States). Briefly, the paraffin sections were deparaffinized, rehydrated, and incubated in DNA labeling solution for 60 min at 37°C, followed by incubating in antibody solution for 30 min at room temperature (RT). A 7-AAD/RNase A solution was added, and the sample was incubated for 30 min at RT and then analyzed with fluorescence microscopy.

### Immunohistochemistry Analyses

IHC analyses were performed as follows. Briefly, 4-μm paraffin-embedded sections were subjected to deparaffinization, antigen retrieval, and goat serum blocking. Sections were then incubated with the primary antibody, followed by HRP-labeled secondary antibody (Gene Tech, Shanghai, China), and the sections were visualized with a DAB system (Gene Tech). Anti-mouse IFN-γ, anti-mouse CD34 (Novus Biologicals, Centennial, United States), anti-mouse γ-H2AX, anti-mouse RASSF1A and anti-mouse ERCC1 (Abcam, Cambridge, United Kingdom) were used as the primary antibodies.

### Enzyme-Linked Immunosorbent Assay

C57BL/6 mice from the five groups were treated as indicated. Mice were sacrificed at 8 days, and the same weight of distal duodenal was collected from each mouse. The tissue was homogenized and centrifuged, and the supernatants were collected for further ELISA. The levels of malonic dialdehyde (MDA), glutathione peroxidase (GSH-Px), myeloperoxidase (MPO), and diamine oxidase (DAO) in small intestine tissue supernatants were quantitated with ELISA kits (Neobioscience, Shenzhen, China) according to the manufacturer’s instructions. Each experiment was performed in triplicate at a minimum.

### mRNA and Protein Expression Analysis

Small intestines and treated IEC-6 cells were harvested, and total RNA and protein were extracted with Trizol reagent (Invitrogen, United States) and RIPA lysis buffer (Beyotime Biotechnology, Beijing, China), separately. Real-time RT-PCR was performed on an ABI7500 Real-time PCR Detection System (Life Tech, Thermo Fisher Scientific, United States) with SYBR Premix Ex Taq ™ ^II^ (TaKaRa, Ohtsu, Japan). The primers are listed in Supplementary Table S1. Total protein was extracted and subjected to western blotting analysis. Anti-rat RASSF1A, anti-rat ERCC1 (Abcam, Cambridge, United Kingdom), anti-rat cleaved caspase 3 (Cell Signaling Technology, Danvers, United States), and β-actin antibodies (Abcam, Cambridge, United Kingdom) were used as primary antibodies and then stained with appropriate HRP-conjugated secondary antibody (KPL, Gaithersburg, United States).

### Small Interfering RNA Knockdown of RASSF1A and ERCC1

RASSF1A and ERCC1 synthetic duplexed siRNA oligonucleotides were purchased from GenePharma (Suzhou, China). A validated nonsilencing siRNA and GADPH siRNA (GenePharma) were used as negative and positive controls, respectively. IEC-6 cells were cultured in 6-well plates at 40–50% density on the day of transfection. Cells were transfected with 7 μmol/L siRNA and 5 μL Lipofectamine 3,000 (Invitrogen, United ). At 24 h after transfection, the cells were used to evaluate the radioprotective effect of FSGen on IEC-6 cells. Real-time PCR and western blot analysis were employed to analyze the gene knockdown efficiency. The experiment was performed in triplicate at a minimum.

### Statistical Analysis

For statistical analysis, data obtained from at least three independent experiments were evaluated using GraphPad Prism software version 6 (GraphPad software, San Diego, United States). Survival was analyzed by the log-rank test. Statistical significance was determined using unpaired two-tailed Student’s t-test or analysis of variance (ANOVA) as indicated in the legend (^∗^ *p* < 0.05, ^∗∗^ *p* < 0.01, ^∗∗∗^ *p* < 0.001, and ^∗∗∗∗^ *p* < 0.0001). Flow cytometry data were analyzed using FlowJo v10. The number of sampled units, n, is indicated in the figure legends.

## Results

### Extraction, Purification and Identification of Genistein From *Fructus sophorae*

**FIGURE 1:**
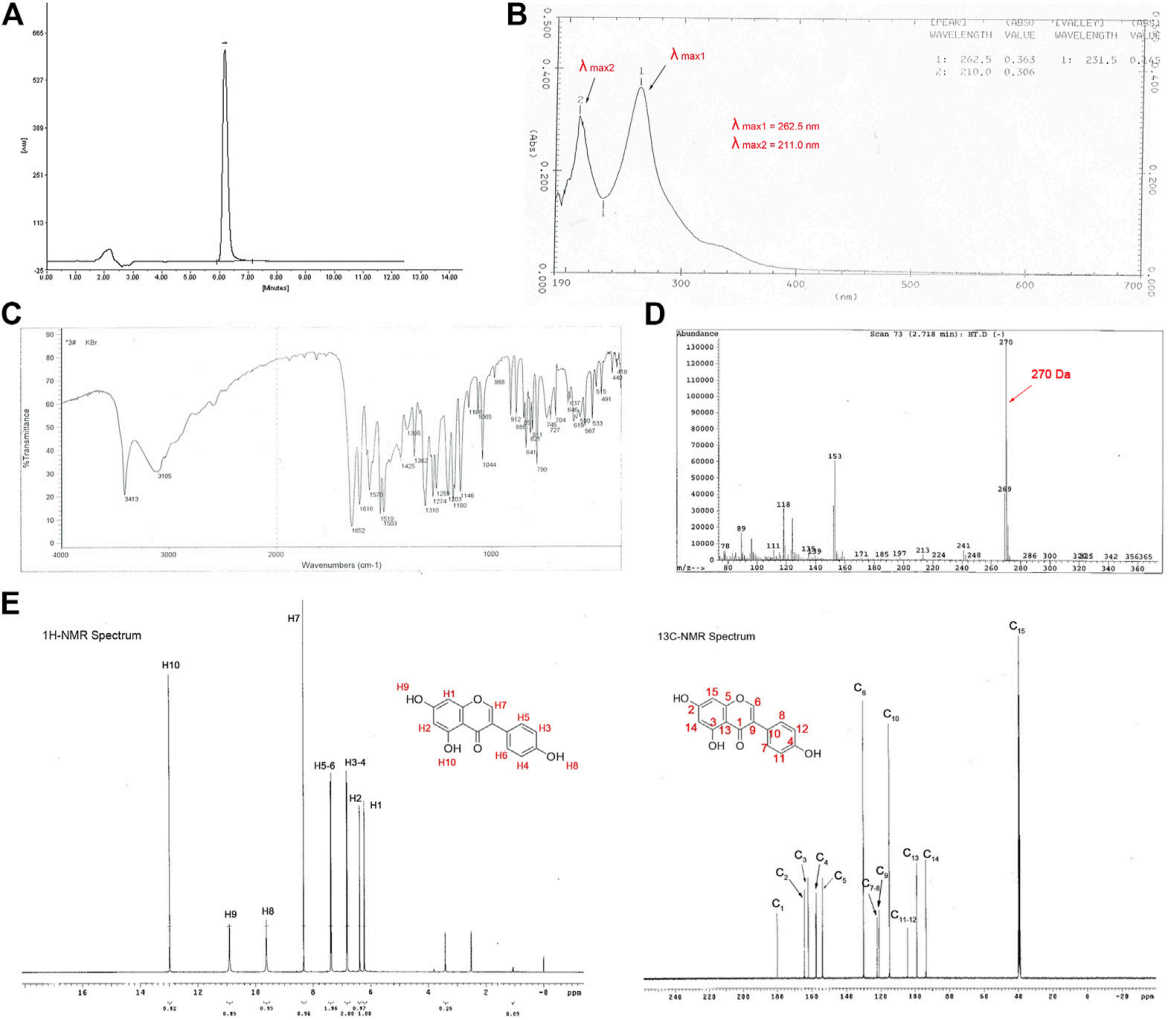
Identification of the extract from *Fructus sophorae*. HPLC, IR, UV, NMR, and MS showed that the extract possessed the structure of genistein. **(A)** HPLC chromatograms of hydrolysate from *Fructus sophorae*, with a hydrolysate purity of 98.82%. **(B)** UV spectra of the extract showed two absorption peaks at 211.0 and 262.5 nm. **(C)** IR spectra of the extract indicated that the ø-CO, ø-OH, and phenyl structure. **(D)** MS of the extract showed that the molecular ions of the sample are 270, and the degree of unsaturation is 11. **(E)** ^1^H **(left)** and ^13^C NMR **(right)** spectra of the extract showed 8 and 13 proton peaks, respectively (except control peaks).

The air-dried and powdered *Fructus sophorae* was extracted with hot alkaline water at a pH of 8.5. After filtration and evaporation, the alkaline water extract was partitioned with concentrated hydrochloric acid, water and EtOH, cleaned via pulp filtration, and evaluated by HPLC. In this study, a total of three batches were extracted, and the example HPLC chromatogram of the sample is shown in Figure 1A. As expected, the purity and percent content of the three batches met the criterion of “Guide of New Drug and Chinese Medicine”. The purity of the three batches was over 98% (98.82, 100, and 98.36%), with an average of 99.06 ± 0.69%. The percent content was 98.96, 99.71, and 98.13%, with an average of 98.93 ± 0.65%. The standard genistein curve was calculated by the peak area (*Y*) and the concentration of the working solutions (*X*). The linear regression equation and the correlation coefficient were *Y* = 2.178*X*+0.017 and r = 0.9998, respectively. The concentrations of the three batches were 67.68864, 68.20164, and 67.12092 μg/ml.

The extract structure was determined on the basis of spectroscopic evidence (MS, UV, IR, ^1^H NMR, and ^13^C NMR). The extract possessed the molecular formula C_15_H_10_O_5_ with eleven degrees of unsaturation, as revealed by MS analysis (*m/z* 270) (Figure 1D). The UV absorption maxima were 211.10 and 262.5 nm, characteristic of aromatic heterocyclic B and E bands, respectively (Figure 1B). The IR spectrum showed absorption bands for hydroxyl groups (3,411 cm^−1^), carbonyl groups (1,652 cm^−1^), and phenyl rings (811, 822, 1,615, 1,570, 1,520, and 1,503 cm^−1^) (Figure 1C). The ^1^H NMR spectrum showed eight proton peaks (δ6.23, δ6.39, δ6.83, δ7.39, δ8.33, δ9.63, δ10.91, and δ12.98) (Figure 1E, left). The ^13^C NMR spectrum exhibited 13 peaks corresponding to 15 carbons in the molecule (Figure 1E, right). In conclusion, the extract spectra data was in agreement with the chemical structure of genistein (Sigma-Aldrich) (Supplementary Figure S1). Thus, the extract was deduced as 5,7,4′-trihydroxy isoflavone, which possessed the structure of genistein and was named FSGen.

### Genistein Extracted From *Fructus sophorae* Increases the Viability of IEC-6 Cells After Irradiation

**FIGURE 2:**
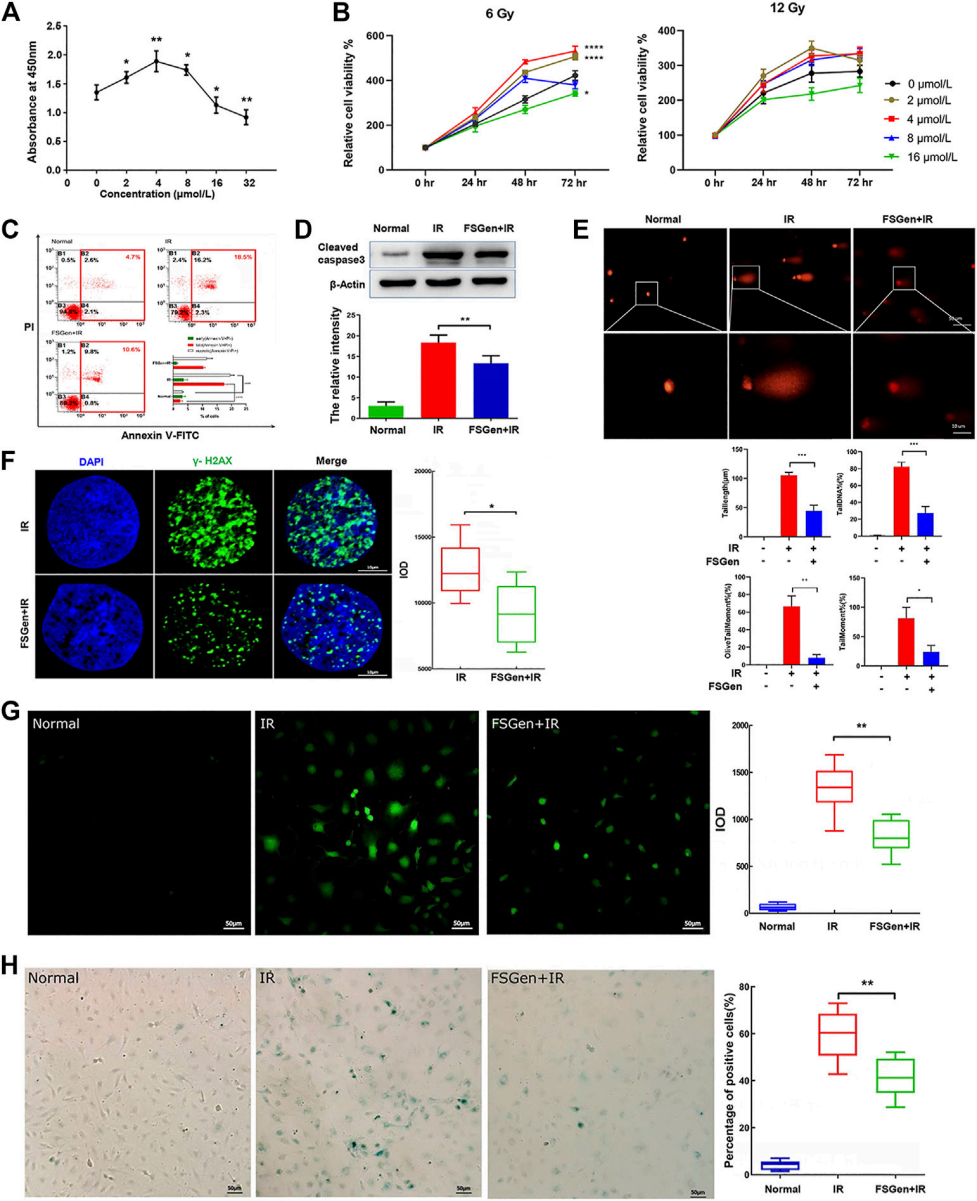
FSGen protects IEC-6 cells from irradiation. **(A)** Cell Counting Kit-8 at 48 h after treatment by different concentrations of FSGen (0, 2, 4, 8, and 16 μmol/L). **(B)** Effect of different concentrations of FSGen (0, 2, 4, 8, and 16 μmol/L) on cell viability 24, 48, and 72 h after 6 Gy **(left)** or 12 Gy **(right)** X-ray irradiation. Cell viability was determined using the CCK-8 assay. **(C–H)** Vehicle (DMSO) or 4 μmol/L FSGen was added to IEC-6 cells for 24 h before 6 Gy of X-ray irradiation. After a further 72 h of culture, apoptosis was analyzed by flow cytometry **(C)** and by detecting apoptosis-related caspase 3 level **(D)**. DNA damage was analyzed by SCGE **(E)** and by γ-H2AX staining **(F)**. The generation of ROS and senescence was determined by DCF-DA assay **(G)** and by β-galactosidase staining **(H)**. Measurement were conducted in triplicate. Bar graphs represent mean ± SD (*n* = 3). ∗*p* < 0.05, ∗∗*p* < 0.01, ∗∗∗*p* < 0.001.

The protective effects of phytochemicals against irradiation are closely related to concentration. Accordingly, we performed CCK-8 assay to elucidate the role of FSGen in IEC-6 cells. 2–8 μmol/L FSGen led to an increased proliferation of IEC-6 cells, whereas 16 μmol/L FSGen led to an decreased proliferation (Figure 2A). Consistently, FSGen show significantly protective effect to IEC-6 cells after 6 or 12 Gy irradiation after 48 h treatment (Figure 2B). 4 μmol/L FSGen showed the greatest protection on IEC-6 cells viability after 6 Gy irradiation. The cell viability decreased in the high-concentration FSGen-treated groups (8 and 16 μmol/L) compared with 4 μmol/L-treated groups. However, the cell viability of the 12 Gy irradiation group was lower than that of the 6 Gy group. These results indicated that the radioprotective bioactivity of FSGen was closely related to its concentration, irradiation dose, and time phase. Based on this result, we chosen 6 Gy X-ray irradiation dose and 4 μmol/L FSGen in the following cell experiments.

### Genistein Extracted From *Fructus sophorae* Reduces Irradiation-Induced IEC-6 Cell Apoptosis

IR-induced cytotoxicity commonly leads to cell apoptosis. By using the Annexin V/PI staining assay, we showed that the apoptotic cell percentage of vehicle-pretreated group and the FSGen + IR group (10.6 and 18.5%, respectively), was significantly different (Figure 2C). Additionally, we detected cleaved caspase 3 protein expression, which is a marker of apoptosis, to confirm the apoptotic cell death induced by irradiation. The results showed that cleaved caspase 3 was significantly increased after irradiation but decreased in the FSGen + IR group compared with the IR group (Figure 2D). These results demonstrated that 4 μmol/L FSGen could effectively inhibit irradiation-induced apoptosis and thereby showed bioactivity to alleviate irradiation damage.

### Genistein Extracted From *Fructus sophorae* Prevents Irradiation-Induced IEC-6 Cell DNA Damage

Radiation-induced cells and tissue damage are usually triggered by ROS and DNA damage. Denatured cleaved DNA fragments have the ability to migrate out of the cell under the influence of an electric potential, whereas undamaged DNA remains within the confines of the cell membrane (Vignard et al., 2013). Based on this principle, the SCGE comet assay, which is a sensitive and reliable method for detecting double and single-strand DNA breaks, was introduced. In this study, 4 μmol/L FSGen or DMSO was applied 24 h prior to 6 Gy irradiation; over a 48 h incubation, we monitored the frequency of comet cells (cells with DNA damage), the length of the comet tail, the tail moment, the olive tail moment and the percentage of DNA in the tail (Figure 2E). The comet formation in the FSGen + IR group was notably lower than the IR group (Figure 2E). Additionally, the results obtained for the comet tail length, tail moment, olive tail moment and percentage of DNA in the tail showed trends similar to the comet incidence. As shown in the right panel in Figure 2E, the tail length of the IR group was 99.4 ± 2.5 μm, which was longer than that of the FSGen + IR group, in which the tail lengths were reduced by 52.7%. The percentage of DNA in the tail was markedly reduced in the FSGen + IR group compared with the IR group, with a reduction rate of 38.1%. The tail moment of the FSGen + IR group was dramatically reduced by 68.6% compared with that of the IR group. The olive tail moment was markedly reduced in the FSGen + IR group compared with the IR group.

Double strand breaks (DSBs) are the primary cytotoxic lesions caused by ionizing radiation (Vignard et al., 2013). We further measured the protective effect of FSGen on DSBs by immunostaining for phosphorylated H2AX (γ-H2AX), which is a sensitive marker of DSBs. Compared with the IR group, the FSGen + IR group significantly had a reduced number of γ-H2AX foci and decreased IOD (Figure 2F). These data strongly indicated that FSGen effectively attenuates DNA irradiation damage to IEC-6 cells.

### Genistein Extracted From *Fructus sophorae* Protects IEC-6 Cells From the Generation of ROS and Senescence After Irradiation

Next, we measured intracellular ROR generation by performing a DCF-DA assay at 48 h after irradiation (Figure 2G). The generation of ROS from the FSGen + IR group was markedly reduced compared with that of the IR group, suggesting that FSGen possessed radical scavenging activity. Similar to the trends of apoptosis, DNA damage, and ROS generation, treatment with 4 μM FSGen before irradiation resulted in a significant decrease in the percentage of senescent cells, with an 18.6% reduction (Figure 2H). These results demonstrate that FSGen confers radioresistance to IEC-6 cells.

### Genistein Extracted From *Fructus sophorae* Potently Protects Against Ionizing Radiation-Induced Lethal Injury

In the pilot study, remarkable mortality was noticed in the group exposed to the 11.5 Gy, where 7.5 and 3.5 Gy mice were still alive on days 10. On the other hand, irradiation of 7.5 Gy leading to mortality within 20 days post-WBI, while under 3.5 Gy, 80% of mice were still alive on days 20. Thus, the following study was performed using the 7.5 Gy rather than the 3.5 Gy to better establish the IR-induced injury.

**FIGURE 3:**
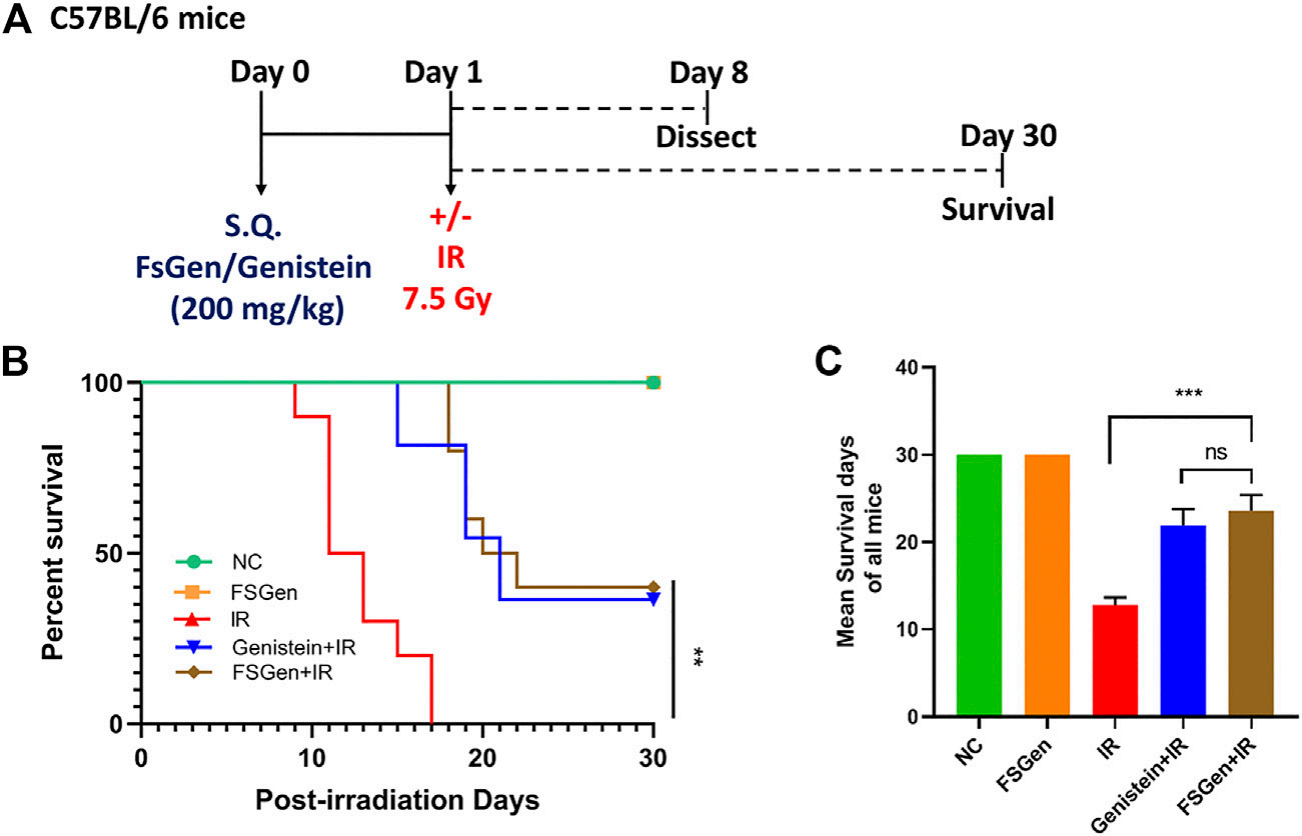
FSGen improves mouse survival after radiation. Mice were pretreated with vehicle (PEG400), genistein (4 μmol/L) or FSGen (4 μmol/L) and subjected to 7.5 Gy irradiation. **(A)** Schematic representation of the mice experiment design used in this study. **(B)** Kaplan-Meier survival curve of mice subjected to 7.5 Gy irradiation at 30 days (mean ± SD, *n* = 7). *p* value was calculated using Log-rank text. **(C)** Mean survival of all mice 30 days after 7.5 Gy irradiation (mean ± SD, *n* = 7). *p* values were calculated using two-tailed *t*-test.^∗∗∗^ *p* < 0.001, ^∗∗∗∗^ *p* < 0.0001, ns, not significant.

To determine the effect of FSGen on IR-induced lethal injury, we first monitored the survival of mice after 7.5 Gy WBI. Mice were pretreated with FSGen, genistein, or vehicle 24 h before irradiation (Figure 3A). FSGen showed good safety through the total 30 days. All vehicle-treated mice (IR group) died 9–20 days after treatment, with an average survival of 11.8 days. In contrast, 50% of mice in the FSGen-treated group survived at least 30 days after treatment, with an average survival of 22.2 days. No significant difference was found in survival rate between the genistein + IR and FSGen + IR group exposed to 7.5 Gy WBI (Figures 3B,C). These data showed that, as the same with genistein, FSGen significantly prolonged the survival of mice after 7.5 Gy WBI.

### Genistein Extracted From *Fructus sophorae* Improves the Outcome of Ionizing Radiation-Induced Gastrointestinal Injury

**FIGURE 4:**
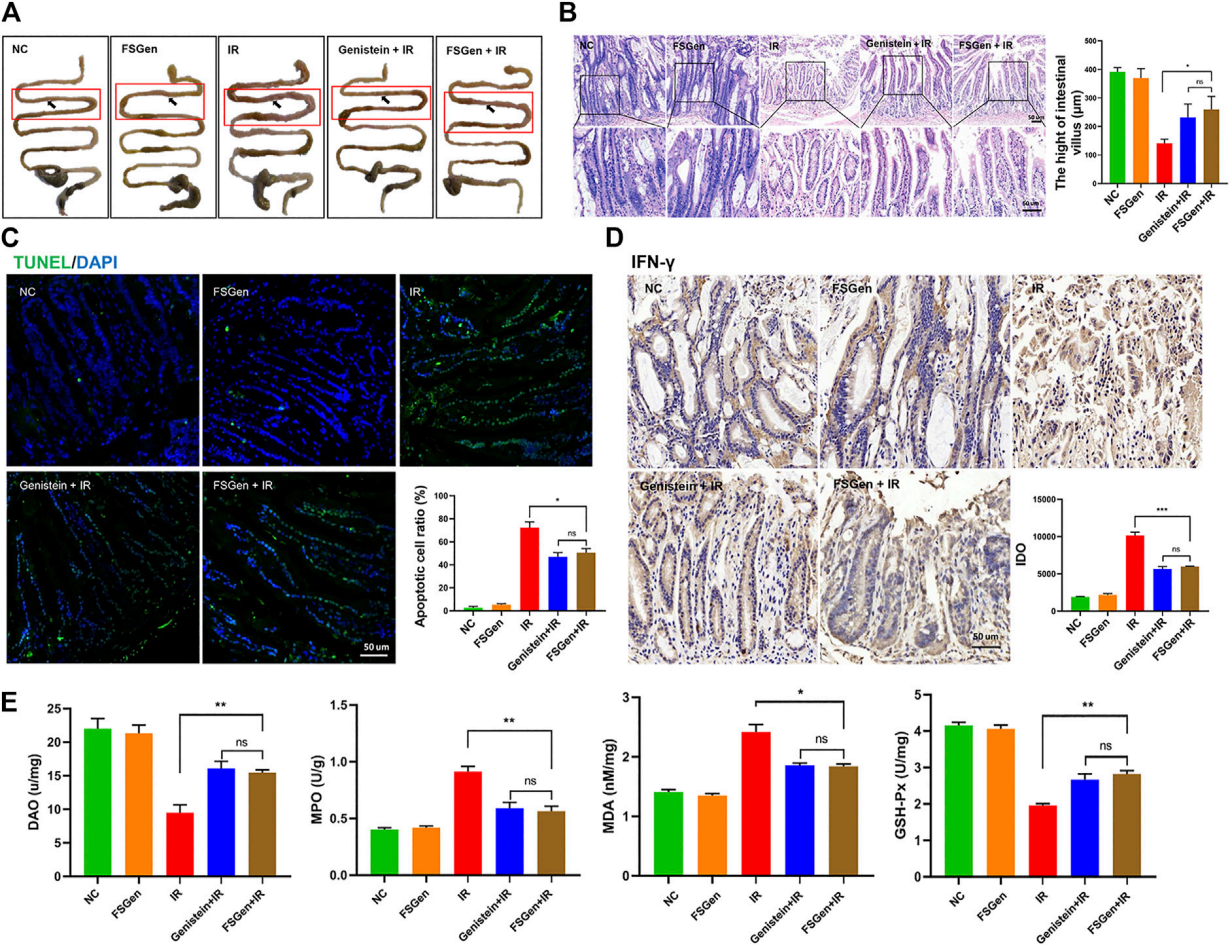
FSGen improves the outcome of radiation-induced GI syndrome. Mice were pretreated with vehicle (PEG400), genistein (4 μmol/L) or FSGen (4 μmol/L) and subjected to 7.5 Gy irradiation. **(A)** Representative images of the morphology of small intestines. **(B)** H&E-stained intestinal tissues from mice **(left)**. Villus height was measured **(right)** (mean ± SD, *n* = 7). **(C)** TUNEL-staining of intestinal tissues from mice. **(D)** IHC IFN-γ staining of mice intestine tissues. **(E)** Protein levels of MDA, GSH-Px, MPO, and DAO were measured by ELISA in each group. MDA, malonic dialdehyde; GSH-Px, glutathione peroxidase; MPO, myeloperoxidase; DAO, diamine oxidase. Bar graphs represent mean ± SD (*n* = 7). *p* values were calculated using two-tailed *t*-test. ^∗^ *p* < 0.05, ^∗∗^ *p* < 0.01, ^∗∗∗^ *p* < 0.001, ns, not significant.

To understand the mechanisms of the radioprotective effect of FSGen on IR-induced injury, we dissected mice and observed the morphology of the intestine and villus after 7.5 Gy WBI. Mice were pretreated with FSGen, genistein, or vehicle 24 h before irradiation and then sacrificed 8 days after irradiation. The effects of FSGen on the morphology of the small intestine are presented in Figure 4A. The morphology of the small intestine, the enterocyte apoptosis, and the inflammation of the small intestine in FSGen group was similar to NC group, which indicated the good safety of FSGen in mice (Figure 4). Compared with the vehicle, FSGen can attenuate IR-induced intestinal wall edema and vasculature congestion. The FSGen group also displayed better preserved villus structure and taller villi ( Figure 4B ). This observation was correlated with an approximately 1.5-fold decrease in the apoptosis of villous cells (Figure 4C) and a significant increase in intestinal DAO levels (Figure 4E, left), as measured by TUNEL staining and ELISA assay, respectively. Compared with vehicle-pretreatment, FSGen pretreatment significantly decreased intestinal IFN-γ (Figure 4D) and MPO levels but increased MDA and GSH-Px protein levels in the intestine (Figure 4E, right). Furthermore, the protective effect of the intestinal of FSGen was in accordance with the genistein (Figure 4). Therefore, FSGen effectively relieves intestinal inflammation reactions and increases the antioxidant capacity of the intestine. These data demonstrated that FSGen potently prevents IR-induced GI injury.

### Genistein Extracted From *Fructus sophorae* Protects Mouse Bone Marrow From Irradiation-Induced Damage

**FIGURE 5:**
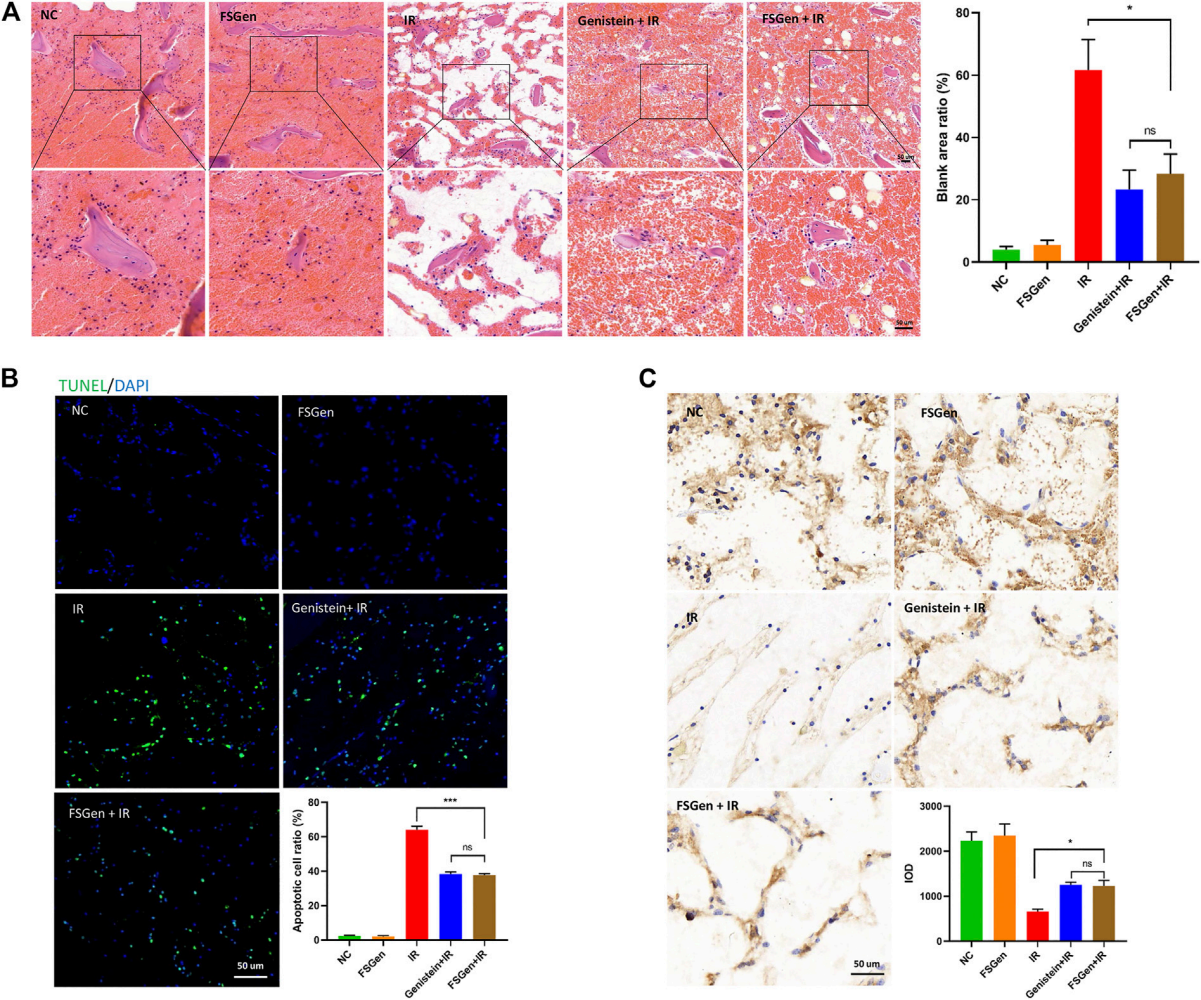
FSGen protects mouse bone marrow from irradiation-induced toxicity. Mice were pretreated with vehicle (PEG400), genistein (4 μmol/L) or FSGen (4 μmol/L) and subjected to 7.5 Gy irradiation. The sterna was analyzed at 8 days. **(A)** H&E-stained sterna from mice **(left)**. **(B)** TUNEL-staining of sterna from mice. **(C)** IHC CD34 staining of mice sterna. Bar graphs represent mean ± SD (*n* = 7). ^∗^ *p* < 0.05, ^∗∗∗^ *p* < 0.001, ns, not significant.

Next, we evaluated the pathological changes in bone marrow from the sectioned sterna after 7.5 Gy WBI. Marrow cellularity was estimated in microscopic fields. Irradiation significantly reduced bone marrow cellularity, whereas the FSGen-pretreated group showed marked hematopoietic recovery with a significant increase in cellularity on day 7 post irradiation (Figure 5A). In addition, the effect of FSGen on hematopoietic recovery was also showed by the significant reduction in apoptotic cells and the increase of hematopoietic stem cells (CD34-positive cells) in FSGen-pretreated mouse bone marrow on day 7 post irradiation (Figures 5B,C). Consistent with the intestinal results, FSGen showed good safety, and similar protective effect to the bone marrow with the genistein (Figure 5).

### Genistein Extracted From *Fructus sophorae* Regulates the Expression of RASSF1A and ERCC1

**FIGURE 6:**
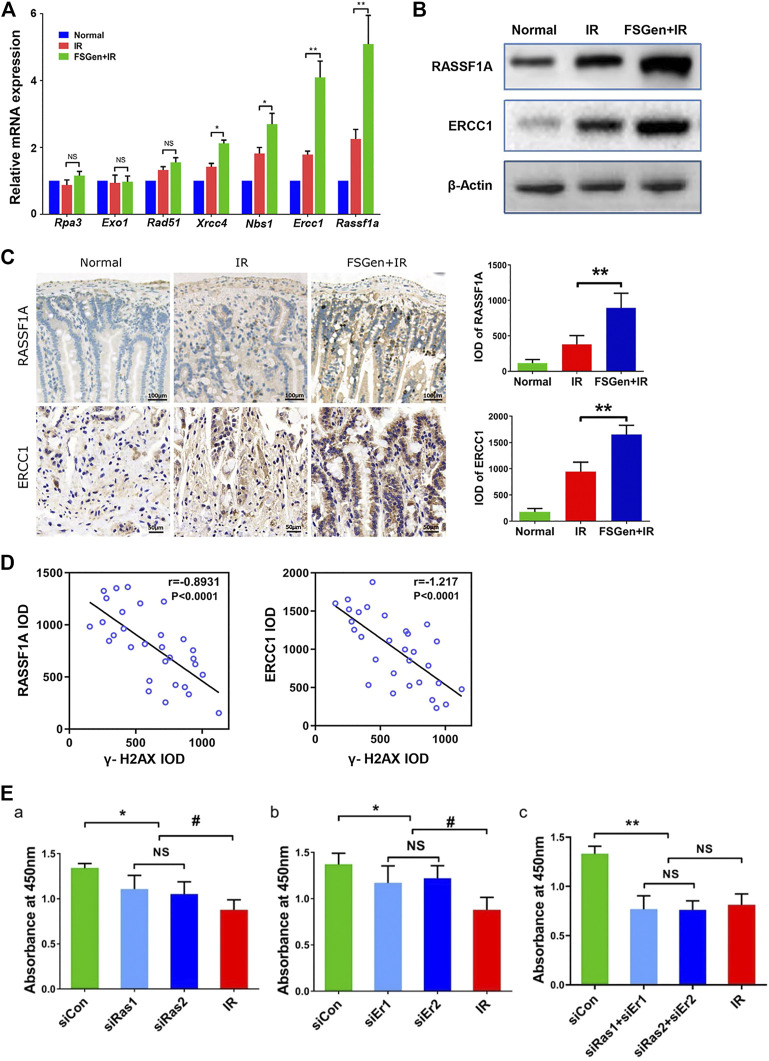
FSGen improves DNA damage repair by upregulating RASSF1A and ERCC1. IEC-6 cells were treated with FSGen for 48 h. **(A)** mRNA expression of DNA repair pathway-related genes were analyzed by Real-time PCR (two-tailed Student’s t-test, mean ± SD, *n* = 7). **(B)** RASSF1A and ERCC1 proteins were analyzed by Western blot. **(C)** IHC RASSF1A and ERCC1 staining of mice intestine tissues. (two-tailed Student’s t-test, mean ± SD, n = 7). **(D)** Correlation analysis between RASSF1A with γ-H2AX and ERCC1 with γ-H2AX in the mouse intestine. Mice were pretreated with vehicle (IR) or FSGen (FSGen+IR) and subjected to 7.5 Gy irradiation (Spearman’s rank correlation test, *n* = 7). **(E)** Bar graph showing the effect of silencing Rassf1a or/and Ercc1(siRas, siEr, siRas+siEr) on cell viability of pretreated the cells with FSGen and irradiation. (two-tailed Student’s t-test, mean ± SD, *n* = 7). Cell viability was determined using the CCK-8 assay. A minimum of three independent runs was performed. ^∗^ *p* < 0.05, ^∗∗^ *p* < 0.01, ns, not significant.

Since the above study strongly indicated that FSGen effectively attenuates DNA irradiation damage in the bone marrow and the intestine, related genes in the DNA repair pathway were evaluated by quantitative real-time PCR. *Rassf1a* and *Ercc1* were the most upregulated genes in the FSGen + IR group (Figure 6A). This upregulation of *Rassf1a* and *Ercc1* was confirmed by western blot analysis (Figure 6B). Moreover, RASSF1A and ERCC1 protein expression in the small intestine was dramatically increased in the FSGen + IR group compared with the IR group (Figure 6C). The expression of RAFF1A and ERCC1 also showed a remarkable negative correlation with γ-H2AX foci in the FSGen + IR and IR mouse groups (Figure 6D).

To determine whether RASSF1A and ERCC1 are required for the radioprotective effect of FSGen, we next sought to investigate whether the inhibited RASSF1A and ERCC1 expression resulted in a decrease in cell proliferation after irradiation. We treated IEC-6 cells with small interfering RNA (siRNA) to knock down RASSF1A and ERCC1. The efficiency of RASSF1A and ERCC1 siRNAs was verified by real-time PCR and Western blot (Supplementary Figure S2), and we randomly used RASSF1A and ERCC1 siRNA1 in all the subsequent experiments. IEC-6 cells were pretreated with 4 μmol/L FSGen or DMSO for 24 h followed by 6 Gy irradiation after RASSF1A and/or ERCC1 siRNA knockdown. We found that the silencing of RASSF1A and/or ERCC1 significantly compromised cell proliferation (siCon vs. siRas/siEr/siRas + siEr), 4 μmol/L FSGen pretreatment showed no radioprotective effect against irradiation (Figure 6E). These data strongly suggest a pivotal role for RASSF1A and ERCC1 in the protective effect of FSGen.

## Discussion

Radiation remains one of the most widely used cancer treatments, but there is no FDA-approved agent for the prevention or treatment of the radiation-induced intestinal injury. Genistein, one of the main isoflavone compounds, has consistently drawn widespread attention for its bioactivity in alleviating radiation damage (Ganai and Farooqi, 2015; Kamran et al., 2016). Our current work established an effective method to extract genistein from traditional Chinese medicine, and it can be administered in a single injection 24 h prior to radiation exposure to prevent GI injury.

Since the major commercially available genistein was chemically synthesized, concerns over chemical residues and high costs have been barriers to clinical application (Fischer et al., 2018). This present study established a method for preparing high-concentration genistein from *Fructus sophorae* extract. *Fructus sophorae*, the dried ripe fruit of *Styphnolobium japonicum* (L.), is widely used in Chinese medicine and is cheap, environmentally friendly and convenient. It is an important step to extract and purify bioactive compounds from natural sources for the application as ingredients and additives in pharmaceutical industries. Due to the simple operation and cheap equipment, batch solvent extraction is the most common and efficiency extracting method in industrial production. The choice of solvent is crucial in a batch solvent extraction process. Many solvents have been used to extract isoflavones, including ethanol, acetone, acetonitrile, and water (Nan et al., 2018). Thanks to the non-toxicity and low cost of ethanol, many processes use it as the extracting solvent for isoflavones extraction (Murphy et al., 2002; Zhang et al., 2007). However, ethanol may also extract a high percentage of lipids, which must be separated by organic solvents such as chloroform. Due to the inherent toxicity, organic solvents like chloroform are limited use in pharmaceutical products (Products, 2019). Thus, it is important to optimize extraction condition and control extraction process. Considering the sophora glycosides can be easy dissolving in hot alkaline water, while the insolubility of lipid components in water, we used a hot alkaline solution (pH 8.5) as the extracting solvent in the present study to skip the organic solvent degreasing step. The purity of the three batches FSGen in this study was 99.06 ± 0.8459, which met the requirements of the set of new drug quality standards. IR, UV, NMR, and MS analysis showed that the FSGen possessed the structure of 5,7,4′-trihydroxy isoflavone. Therefore, with the advantages of saving costs and protecting the environment, our method extracts high purity of FSGen. Since genistein is one of the major active constituents of *Fructus sophorae*, establish the extracting method is important to promote the industrialization of *Fructus sophorae*.

On the basis of extraction, we further evaluate FSGen medicinal activity against radiation. The GI system is an early response organ to radiation. The rapid turnover of intestinal epithelial cells makes the intestinal mucosa especially vulnerable to high radiation exposure during radiotherapy (Moussa et al., 2016). In this study, we first employed rat small intestine IEC-6 cells to evaluate the radioprotective activity of FSGen. FSGen shows the “double effects” when treated IEC-6 cells. The cells exhibited improved viability when incubated with 4 or 8 μmol/L of FSGen after 6 or 12 Gy of X-ray irradiation, while decrease cell viability at a dose up to 16 μmol/L. This finding is consistent with the previous studies of genistein (Song et al., 2015), that genistein protected HL-7702 cells against 6 or 8 Gy X-ray irradiation at a low concentration of 1 and 5 μmol/L, but damage cell viability at a high concentration (20 μmol/L). Although the therapeutic range of FSGen is narrow, fortunately, many pharmacological methods can be used to improve the safety of medicine ingredients. In fact, our finding of FSGen is similar to previous studies of genistein. For instance, Lihua Song, et al. ( Song et al., 2015 ) found that genistein protected HL-7702 cells against 6 or 8 Gy X-ray irradiation at a low concentration of 1 and 5 μM, but damage cell viability at a high concentration (20 μM). Actually, an oral dosage form of FSGen showed in the present study has finished phase I and II clinical trials, which guarantee the safety of this Chinese herbal medicine ingredients (unpublished data).

It is well known that radiation-induced cytotoxicity is triggered by ROS and DNA damage, which commonly leads to cell apoptosis and senescence (Stone et al., 2003). To further investigate the radioprotective effect of 4 μmol/L FSGen on IEC-6 cells, the effects of FSGen against cell apoptosis, DNA damage, ROS generation and cell senescence were also evaluated in our study. The results showed that pretreatment with FSGen resulted in a sharp reduction in the percentage of apoptotic cells and apoptosis-associated cleaved caspase-3 protein levels. The comet assay and γ-H2Ax staining showed a marked protective effect of FSGen on radiation-induced single-strand and double-strand DNA breaks. Moreover, 4 μmol/L FSGen also significantly decreased ROS generation and effectively protected against senescence in IEC-6 cells. Our results suggested that FSGen had radioprotective effect on the small intestine *in vitro*.

Michael Landauer’s group demonstrated radioprotective effects of genistein against Î³-irradiation without toxicity or any apparent side effect in mice receiving a single subcutaneous injection 24 h prior to irradiation (Landauer et al., 2003). Son et al. found that genistein had a protective effect on the intestinal damage induced by irradiation in tumor-bearing mice (Son et al., 2013). Some data showed that genistein could also be an effective radioprotectant against hematopoietic-acute radiation syndrome (Zhou and Mi, 2005; Davis et al., 2007; Landauer et al., 2019). Based on the good safety of FSGen in mice, our data also showed that most FSGen-pretreated and genistein-pretreated mice continued to survive beyond 20 days, while all vehicle-treated mice died before 20 days after 7.5 Gy WBI, which indicates that in agreement with genistein, pretreatment with FSGen could ameliorate radiation-induced tissue toxicity.

Acute injury to the small intestine is generally assessed by changes in the morphology of the intestine and villus height after irradiation. High-dose radiation injury exacerbated mucosal inflammation and dysfunction or bacteremia, which led to the swelling and flushing of the intestinal mucosa and modified the submucosa, resulting in necrosis and exfoliation (Saha et al., 2012). The height of the villi, the ratio of apoptotic cells, and the expression of inflammatory factors may be very sensitive and suitable biodosimetry markers after irradiation. In our WBI mouse model, the FSGen + IR group showed accordance with the genistein + IR group in protecting RI-induced GI injury. Pretreatment with FSGen improved the edema and congestion of the intestine and significantly increased the height of villus, which may be due to the protective function of FSGen against apoptosis and inflammation. A large amount of irradiation damage is caused by the generation of reactive oxygen species. The antioxidant properties of genistein reduce lipid peroxidation and stabilize the cell membrane structure (Rahman Mazumder and Hongsprabhas, 2016). The similar to genistein treatment, FSGen treatment resulted in a significant decrease in MDA levels while increasing GSH-Px levels, suggesting that FSGen protects against radiation-induced intestinal injury via its antioxidative activity. Our results also demonstrated that FSGen effectively protected IR-induced bone marrow toxicity. Bone marrow is a highly radiosensitive tissue. In the current study, consistent with genistein-pretreated mice, mice treated with FSGen exhibited significantly increased bone marrow myeloid cellularity and CD34-positive hematopoietic stem cells after irradiation, while the number of apoptotic cells was decreased in the bone marrow. These findings indicated that FSGen not only reduced various irradiation-associated parameters related to intestinal injury but also promoted mouse bone marrow hematopoietic recovery.

The molecular mechanisms of IR-induced GI protection by FSGen are complex but likely upregulated DNA damage repair genes were identified in expression profiles. Our data revealed a marked difference in the mRNA expression of DNA damage repair genes, in IEC-6 cells between FSGen-pretreated and control groups, especially *Rassf1a* and *Ercc1*. RASSF1A is a tumor suppressor, which regulates several tumor-related signaling pathways and interferes with diverse cellular processes. Previous studies showed that RASSF1A can regulate Ras activation and suppresses oxidative DNA damage and chromosomal damage (Park et al., 2011). It is also phosphorylated by ATM and responding to the DSB damage signals (Hamilton et al., 2009). ERCC1 forms a heterodimer endonuclease complex with XPF. The ERCC1-XPF heterodimer plays a pivotal role in nucleotide excision repair and DBS repair in mammalian cells (Elmenoufy et al., 2019). Our study showed the dramatically upregulated of RASSF1A and ERCC1 in the intestine of FSGen-pretreated mice. Also, these two proteins had a positive correlation with DNA DSBs in FSGen-pretreated and control mice. Furthermore, as expected, knocking down RASSF1A and ERCC1 expression altered cell viability, indicating the key role of the *Rassf1a* and *Ercc1* genes in FSGen-mediated radioprotective activity. Our findings suggested that FSGen-mediated *Rassf1a* and *Ercc1* gene upregulation might play important roles in protecting IR-induced GI injury. However, our study did not clarify the signaling pathways underlying in FSGen regulation of RASSF1A and ERCC1. More future studies directed toward to evaluate the signaling pathways of FSGen will help to further elucidate the beneficial effects of FSGen.

## Conclusion

In conclusion, our work established a method to extract genistein from the traditional Chinese medicine *Fructus sophorae*. Furthermore, we confirmed the extracted genistein has the strong activity that alleviated and protected against IR-induced GI injury and bone marrow toxicity in a WBI mouse model. Since genistein is one of the major active constituents of *Fructus sophorae,* establish the extracting method is important to promote the industrial application of *Fructus sophorae* and can provide a better source for genistein.

## Data Availability

The original contributions presented in the study are included in the article/Supplementary Material, further inquiries can be directed to the corresponding authors.

## Abbreviations

IR, ionizing radiation; GI, gastrointestinal; WBI, whole-body irradiation; FSGen, genistein extracted from Fructus sophorae; API, active pharmaceutical ingredient; HPLC, high-performance liquid chromatography; MS, mass spectrometry; IR, infrared spectroscopy; NMR, Nuclear magnetic resonance spectroscopy; PI, propidium iodide; SA-β-gal, Senescence associated β-galactosidase; NMA, normal melting agarose; LMA, low melting agarose; sub-Q, subcutaneous; IHC, immunohistochemistry; ELISA, enzyme-linked immunosorbent assay; MDA, malonic dialdehyde; GSH-Px, glutathione peroxidase; MPO, myeloperoxidase; DAO, diamine oxidase; siRNA, small interfering RNA; DSB, double-strand breaks.
