# Supplementary material for: Genistein From Fructus sophorae Protects Mice From Radiation-Induced Intestinal Injury
Source: Front Pharmacol. 2021 May 21;12:655652. doi: 10.3389/fphar.2021.655652 (PMC8175795; doi:10.3389/fphar.2021.655652)
Supplement: Supplementary file 1 [file DataSheet1.docx]

**Supplementary**

Table 1. Primers

| Primer name | Primer sequence (5’ – 3’) |
| --- | --- |
| *Rpa3*-F | AGTATATCGAACGACCCGTGTG |
| *Rpa3*-R | CTGTCACCTTCCCGACTACT |
| *Exo1*-F | ATTGCAGCAGTTCCGGAGAA |
| *Exo1*-R | TGCGAGAGGCTTGATGTGTT |
| *Rad51*-F | TACATTGACACCGAGGGCAC |
| *Rad51*-R | GCTTGGTAAAGGAGCTGGGT |
| *Xrcc4*-F | ACTGACGTTCAAGGCCGATT |
| *Xrcc4*-R | CAGCTGCTGGACTTCACTCA |
| *Nbs1*-F | CTTCCAGAAATCTGTGCCCACT |
| *Nbs1*-R | TCTTTCGAGCATGGTGACCTA |
| *Ercc1*-F | AGATCCCCAGCAGGCTCTTA |
| *Ercc1*-R | CGCCTTGTAGGTTTCCAGGT |
| *Rassf1a*-F | ACCTTCCTTCGAAATGACCTGG |
| *Rassf1a*-R | GCTTGGGAAAGATCAGGTGT |
| *β-Actin*-F | AGAGGGAAATCGTGCGTGAC |
| *β-Actin*-R | CATCTGCTGGAAGGTGGACA |


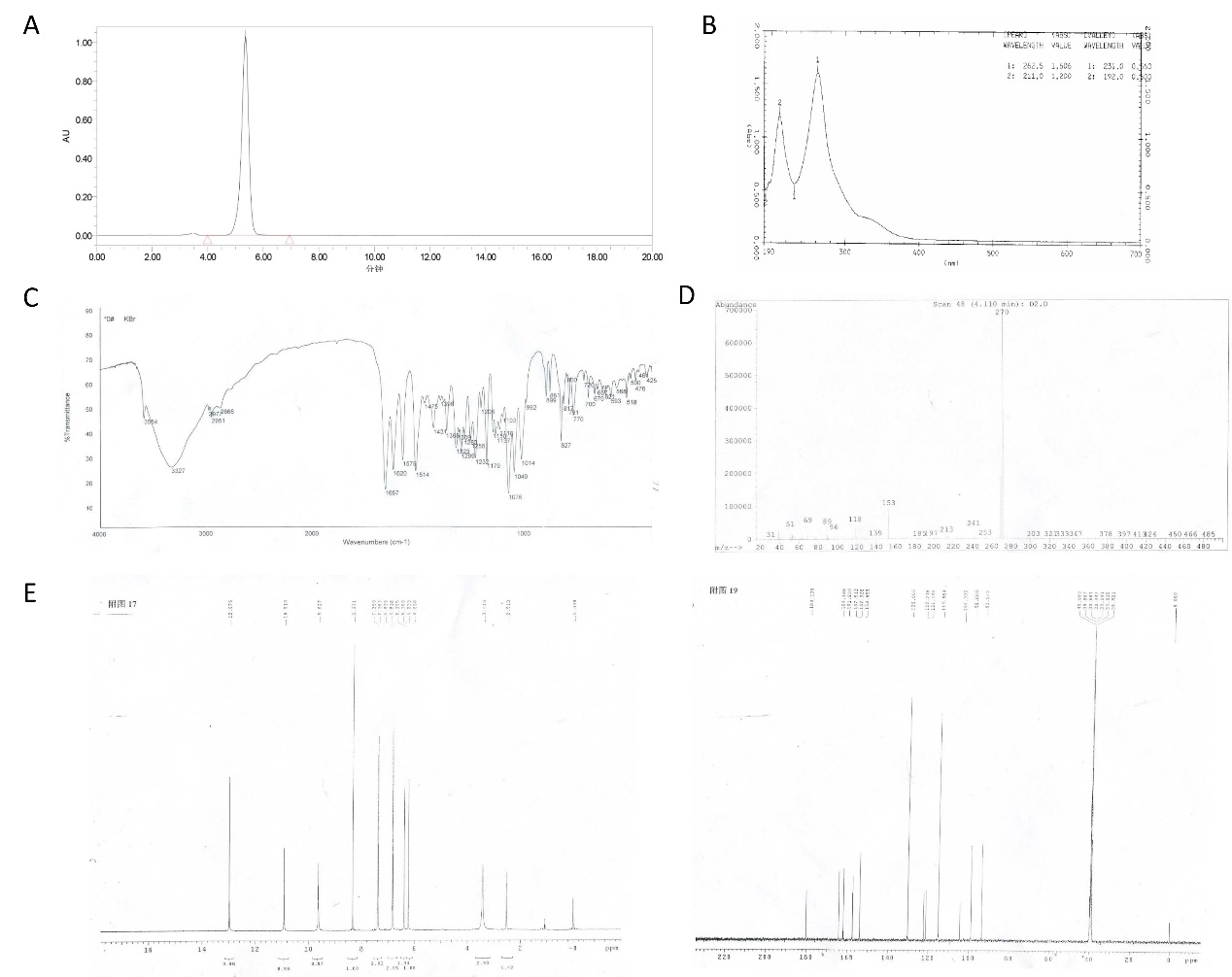


**Supplementary Figure S1**. **The chemical structure of genistein (Sigma-Aldrich)**. A, HPLC chromatograms. B, UV spectra. C, IR spectra. D, Mass spectrometry. E, ^1^H (left) and ^13^C NMR (right) spectra, respectively, (except control peaks).


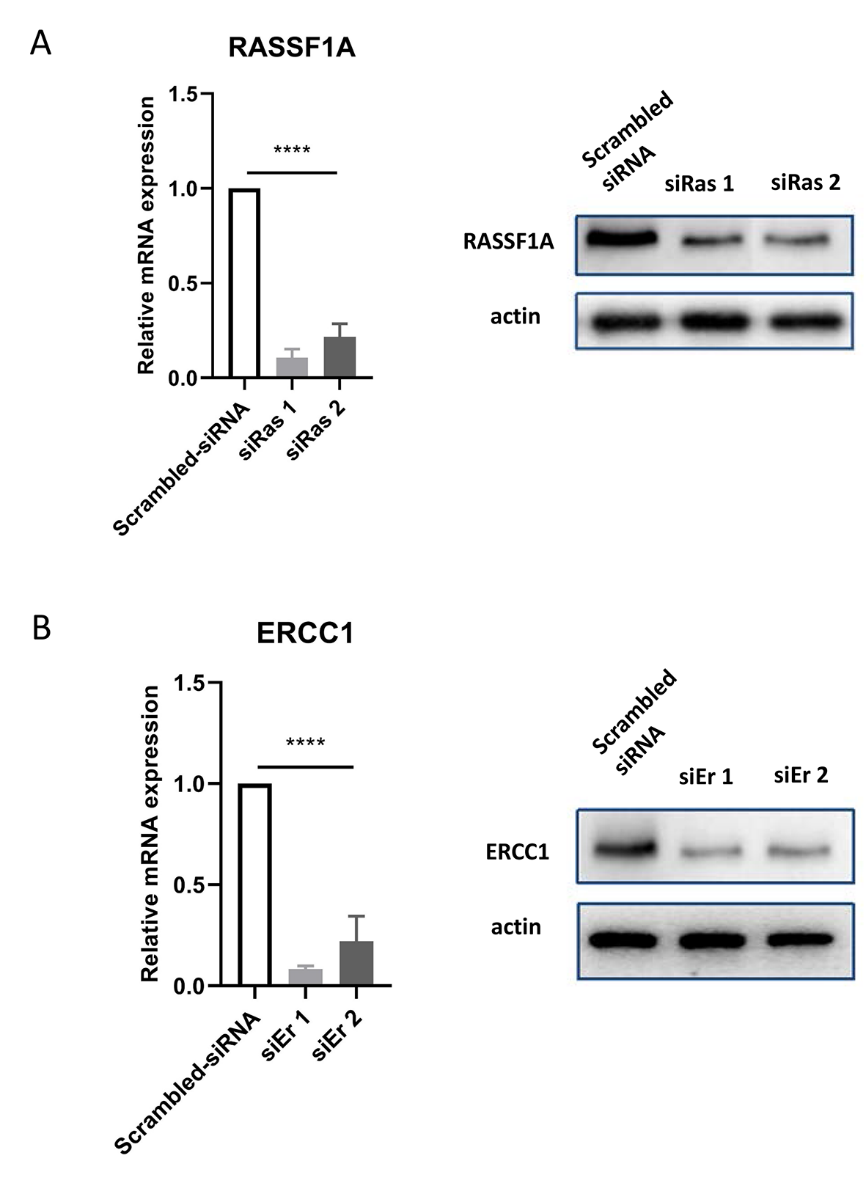


**Supplementary Figure S2**. **Evaluation of RASSF1A and ERCC1 knockdown efficiency by real-time PCR and Western Blot.** (A) The efficiency of RASSF1A knockdown by RASSF1A siRNAs (siRas 1, siRas 2) was verified by real-time PCR and Western Blot. (B) The efficiency of ERCC1 knockdown by ERCC1 siRNAs (siEr 1, siEr 2) was verified by real-time PCR and Western Blot.
